# Supplementary material for: Domain adapted brain network fusion captures variance related to pubertal brain development and mental health
Source: Nat Commun. 2023 Oct 23;14:6698. doi: 10.1038/s41467-023-41839-w (PMC10593774; doi:10.1038/s41467-023-41839-w)
Supplement: Supplementary file 1 — Supplementary Info [file 41467_2023_41839_MOESM1_ESM.pdf]

Domain adapted brain network fusion captures variance related to pubertal brain development and mental health

Dominik Kraft[1], Dag Alnæs[2], Tobias Kaufmann[1, 2, 3]

[1] Department of Psychiatry and Psychotherapy, Tübingen Center for Mental Health, University of Tübingen, Tübingen, Germany
[2] Norwegian Centre for Mental Disorders Research, University of Oslo and Oslo University Hospital, Oslo, Norway
[3] German Center for Mental Health (DZPG), partner site Tübingen, Tübingen, Germany

Address for correspondence:

Dominik Kraft, Dr.
Dominik.Kraft@med.uni-tuebingen.de

Tobias Kaufmann, Prof. Dr.
Tobias.Kaufmann@med.uni-tuebingen.de

Department of Psychiatry and Psychotherapy
Tübingen Center for Mental Health
University of Tübingen
Tübingen, Germany

Inventory of Supporting Information

Supplementary Figures:

Supplementary Fig. 1: Correlation between raw features and first brain embedding for HBN

Supplementary Fig. 2: Associations between brain embeddings and puberty, both in cross-sectional and longitudinal data of the ABCD cohort

Supplementary Fig. 3: Distribution of Δ brain embedding in the ABCD sample stratified for sex and pubertal categories derived from caregivers` reports at 1 year follow-up.

Supplementary Fig. 4: Associations between brain embeddings and dimensional psychopathology, both in cross-sectional and longitudinal data of the ABCD cohort

Supplementary Fig. 5: Distribution of puberty ratings in the ABCD sample stratified for sex, timepoints, and respondent

Supplementary Fig. 6: Distribution of psychopathology measures in the HBN sample as total counts and percentages stratified for sex

Supplementary Tables:

Supplementary Table 1: Pubertal category conversion scheme

Supplementary Table 2: Frequencies of pubertal categories in the ABCD sample.

Supplementary Table 3: Frequencies of psychopathology measures in the HBN sample.

Supplementary Data:

Supplementary Data 1: Association results for HBN (separate file)

Supplementary Data 2: Association results for ABCD (separate file)

Supplementary References

Supplementary Figures

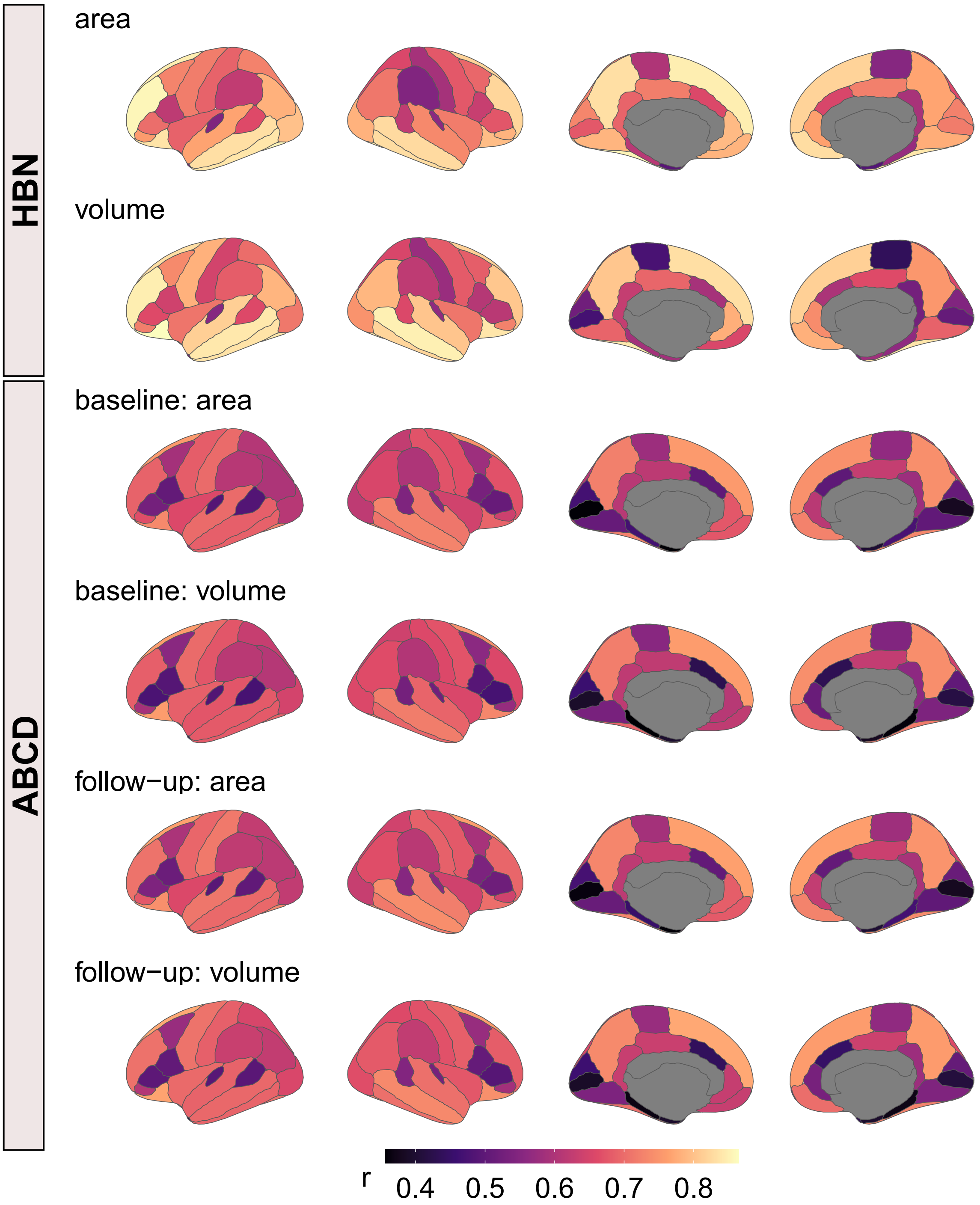

Supplementary Fig. 1: Correlation between raw brain features and first brain embedding. Correlation values indicate Pearson correlation coefficients r. Visualization was performed with ggseg (version 1.6.5 [1]) in R (version 4.2.3). Since algebraic signs of the embeddings might be positive or negative (i.e., akin to an eigenvector), we multiplied negative correlations by (-1) to derive at comparable patterns across cohorts. Spin permutation testing with 5000 iterations indicated significant correlations between brain maps of the ABCD and HBN dataset (all $r > 0.64$, all $p < 0.0004$). Permutations were performed using the ENIGMA Toolbox (version 2.0.3, [2]). $N_{ABCD} = 7776$, $N_{HBN} = 2271$.

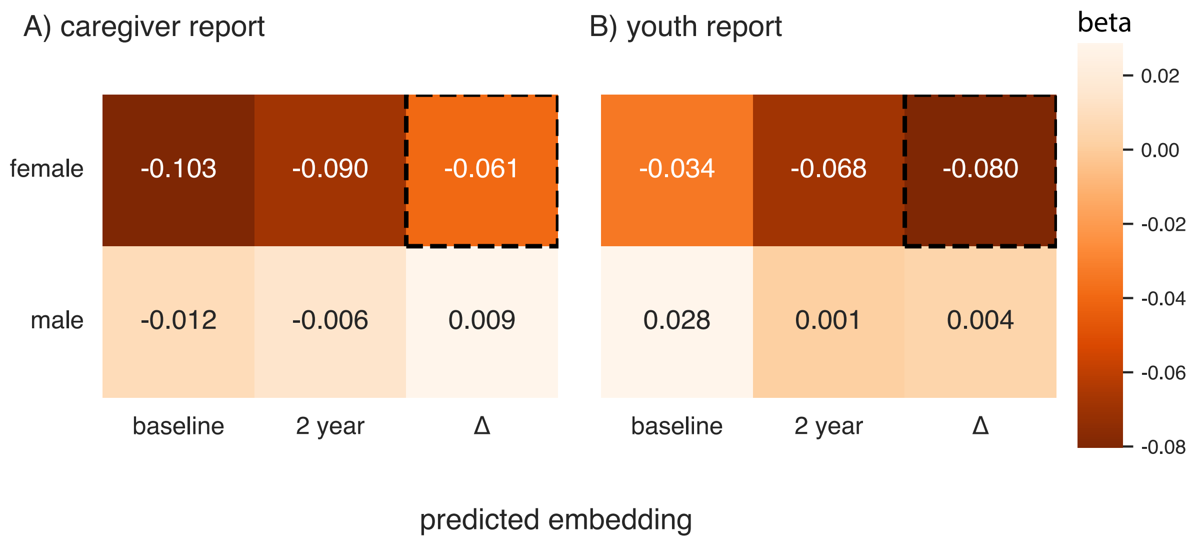

Supplementary Fig. 2: Associations between brain embeddings and puberty, both in cross-sectional and longitudinal data of the ABCD cohort. First two columns in A) and B) refer to associations between predicted brain embeddings and the respective pubertal score (PDS mean) per timepoint. Δ refers to the association between the Δ brain embedding and the ΔPDS mean score. Annotations refer to effect sizes from the linear models and highlighted cells with dashed lines indicate significant results. Linear models were performed in similar vein to the puberty models in the main manuscript but were expanded with Body Mass Index (BMI), Socioeconomic Status (SES), and race/ethnicity as covariates: we ran two cross-sectional models with age, SES, BMI, and race/ethnicity as covariates. Of note for SES there was only baseline data available. Additionally, we ran a longitudinal model in which the delta measures of the brain embedding, the change PDS score and the age and BMI difference between both timepoints were integrated. Moreover, baseline SES and race/ethnicity was added as covariate without a change score. BMI= (height (lb) / height (in)) x 703. Height and weight measures were averaged across two measurements. BMI smaller than 10 or higher than 50 were excluded after manual inspection. Race/ethnicity (ABCD variable acspsw03) refers to 5 different levels: White, Black, Hispanic, Asian, other. Other includes individuals identifying as multiracial and/or belonging to a race/ethnicity with too few members in the sample. For SES variables (i.e., parental education and total family income), “refuse to answer” and “don't know” where encoded as “NaN”. SES variables were transformed via rank-based inverse normal transformation and averaged resulting in a single SES status variable. Exact p-values and effect sizes can be found in Supplementary Data 1. $N_{female\_caregiver}$: baseline= 3338, 2 year= 2784, Δ= 2636 ; $N_{male\_caregiver}$: baseline= 3911, 2 year= 3300, Δ= 3119; N_{female_youth}: baseline= 1476, 2 year= 2752, Δ= 1107 ; N_{male_youth}: baseline= 2261, 2 year= 3425, Δ= 1841.

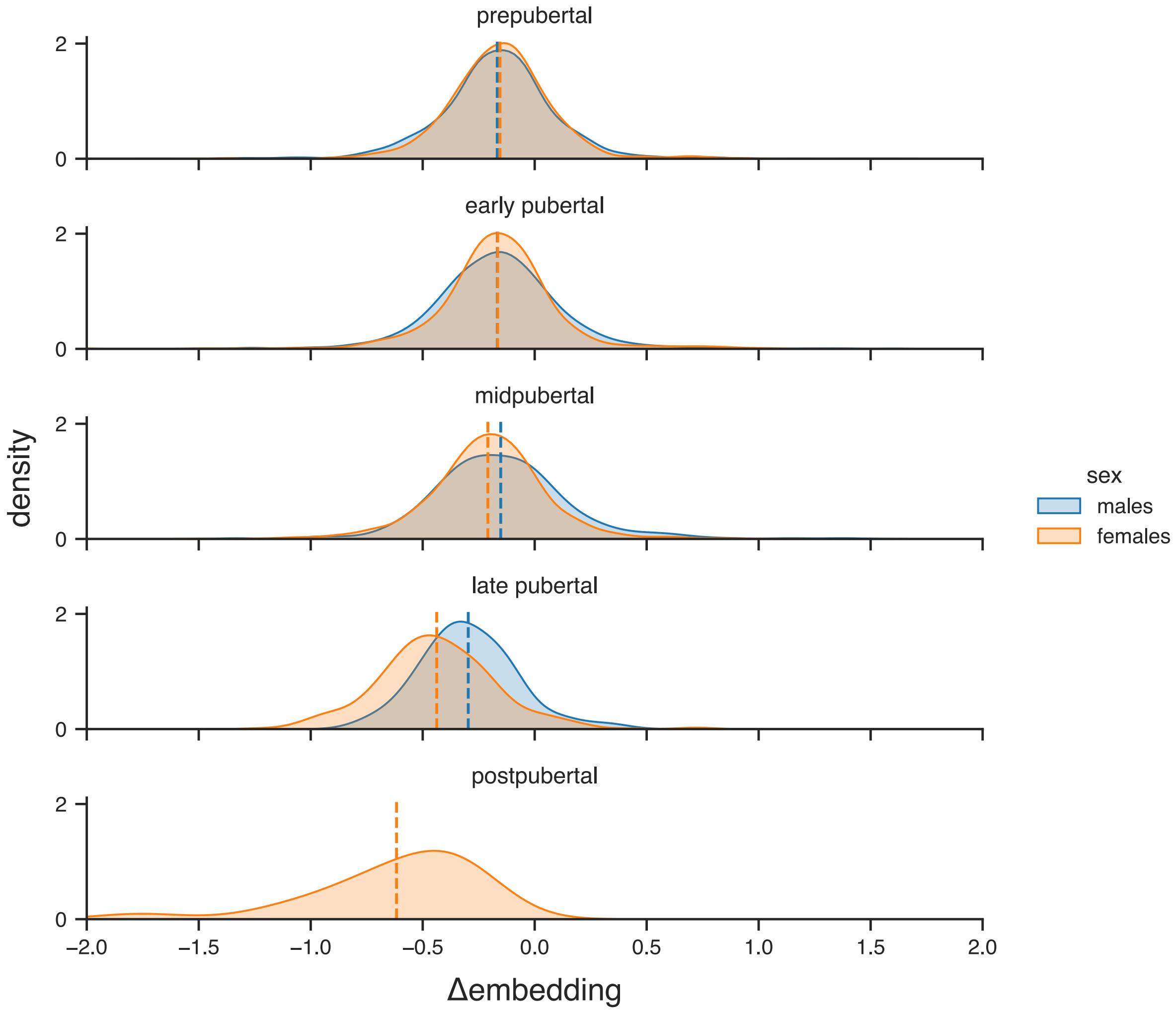

Supplementary Fig. 3: Distribution of Δ brain embedding in the ABCD sample stratified for sex and pubertal categories derived from caregivers` reports at 1 year follow-up. The figure shows the same pattern as Figure 3 (youth report), except that it was based on data from the caregivers` report. Vertical dashed lines indicate the mean Δ brain embedding per group. Note that for the caregiver report there were not enough males in the 'postpubertal' category to allow for density plotting. N= 6770.

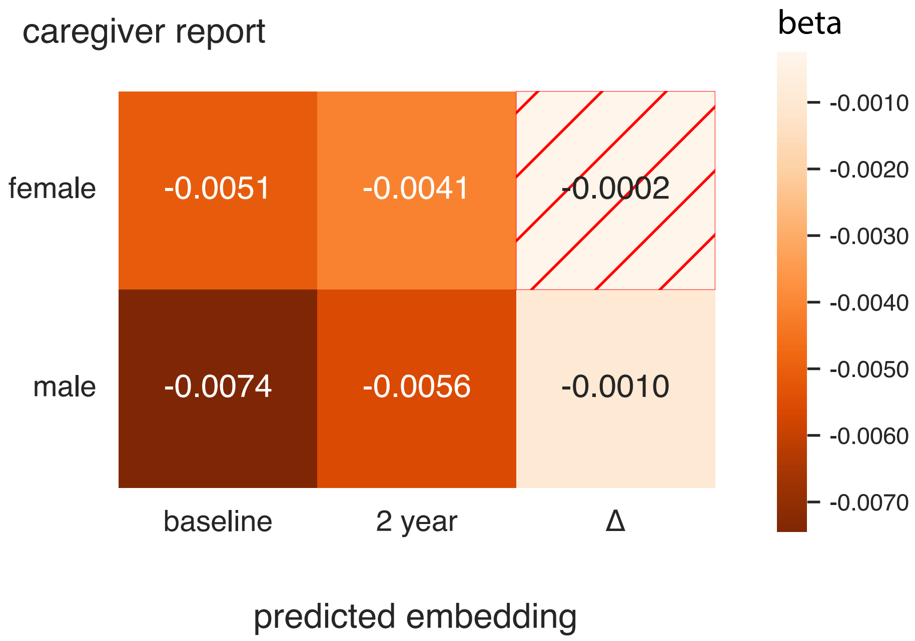

Supplementary Fig. 4: Associations between brain embeddings and dimensional psychopathology, both in cross-sectional and longitudinal data of the ABCD cohort. First two columns refer to associations between predicted embeddings and the respective psychopathology (CBCL Total) per timepoint. Δ refers to the association between the Δembedding and the ΔCBCL score. Annotations refer to effect sizes from linear models and hashed cells indicate non-significant results. Linear models were performed in similar vein to the puberty analyses in the main manuscript: cross-sectional models were calculated with age and site as covariate and the longitudinal model was complemented with site and Δage between both timepoints. Exact p-values and effect sizes can be found in Supplementary Data 1; N_{female}: baseline= 3586, 2 year= 3099, Δ= 3098 ; N_{male}: baseline= 4189, 2 year= 3602, Δ= 3602.

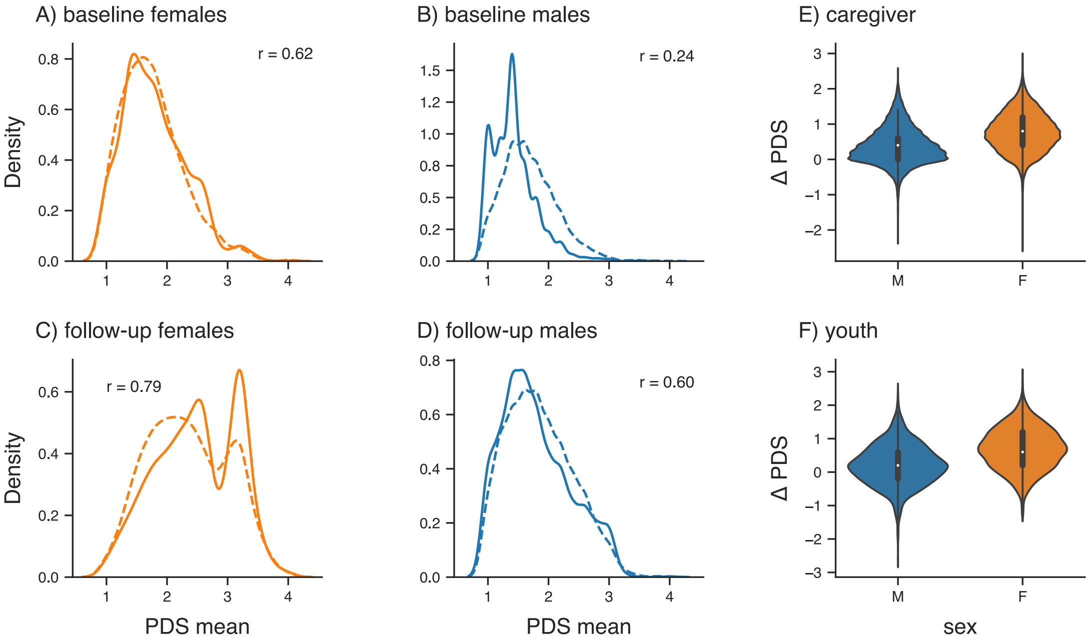

Supplementary Fig. 5: Distribution of puberty ratings in the ABCD sample stratified for sex, timepoints, and respondent (Panel A-D; $N_{female\_baseline}$= 1386, $N_{male\_baseline}$= 2131, $N_{female\_follow\text{-}up}$= 3028, $N_{male\_follow\text{-}up}$ = 3797). Solid lines indicate caregiver and dashed line indicate youth reports. Higher correlation values between puberty ratings at follow-up indicate better alignment between reports from different sources. Panel E and F refer to changes in puberty scores (i.e., ΔPDS) for males ($N_{caregiver}$= 5452, N_{youth}=5451) and females ($N_{caregiver/youth}$=4961). All plots only show data from participants for which data from both timepoints was available. r= Pearson correlation coefficient. M= male, F= female.

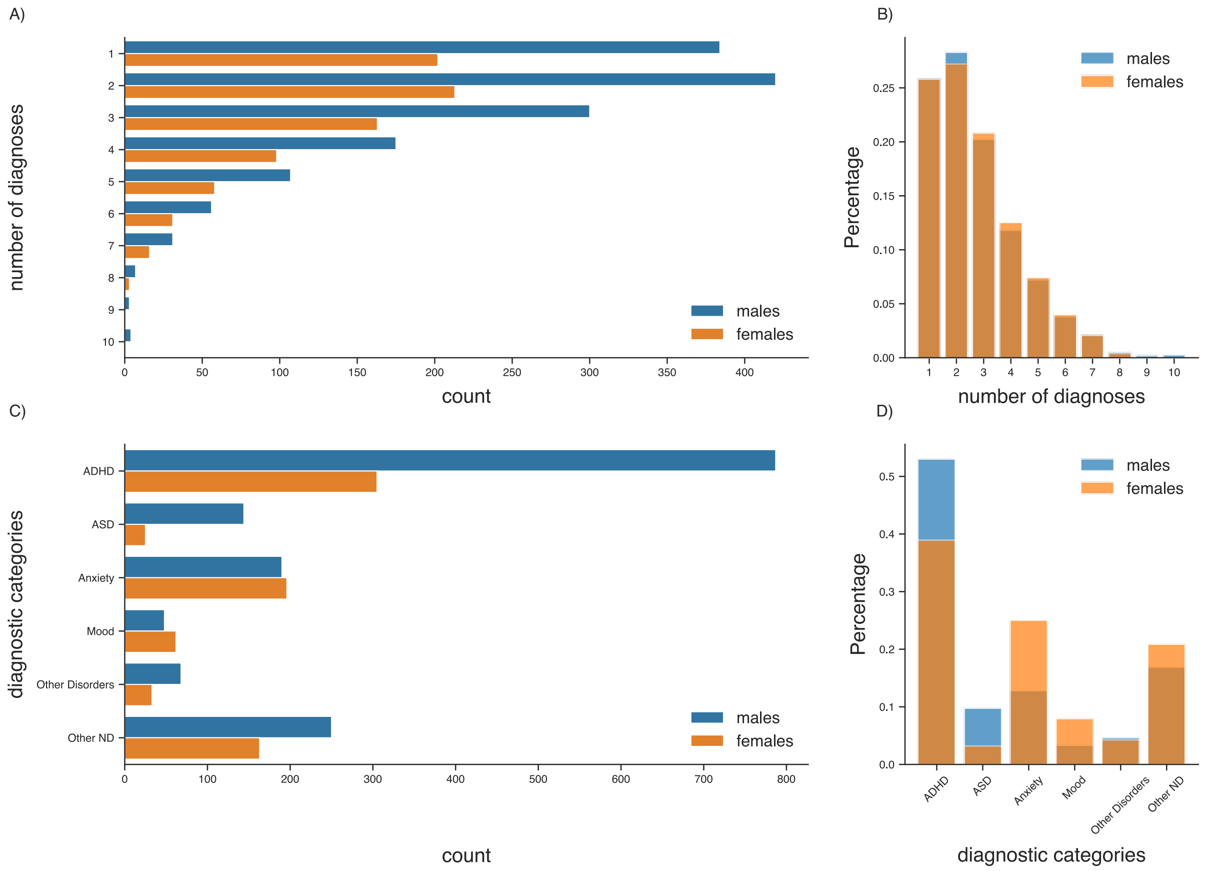

Supplementary Fig. 6: Distribution of psychopathology measures in the HBN sample as total counts (panel A and C) and percentages (panel B and D) stratified for sex. Diagnoses were grouped into six distinct categories following [3]. ADHD= Attention Deficit Hyperactivity Disorder, ASD= Autism Spectrum Disorder, ND= Neurodevelopmental Disorder. N_{HBN} = 2271, see Supplementary Table 3 for additional information about sample sizes per group.

Supplementary Tables

Supplementary Table 1: Pubertal category conversion scheme

sex	conversion scheme	pubertal category
female	premenarcheal + pubertal score = 2	prepubertal
	premenarcheal + pubertal score = 3	early pubertal
	premenarcheal + pubertal score >= 3	mid pubertal
	postmenarcheal + pubertal score <= 7	late pubertal
	postmenarcheal + pubertal score = 8	postpubertal
male	pubertal score = 3	prepubertal
	pubertal score >= 4 and <=5	early pubertal
	pubertal score >= 6 and <=8	mid pubertal
	pubertal score >= 9 and <=11	late pubertal
	pubertal score = 12	postpubertal

Note: Pubertal score refers to the Pubertal Category Score, which depicts the sum of three/two PDS items for male and female, respectively. Male: pubic + facial hair growth + voice deepening. Female: pubic hair growth + breast development. Conversion follows the ABCD variables *'pds_p_ss_female_category' and 'pds_p_ss_male_category'.*

Supplementary Table 2: Frequencies of pubertal categories in the ABCD sample.

Pubertal Category	Caregiver Report		Youth Report	
baseline visit				
	male (N= 4045)	female (N= 3487)	male (N= 3761)	female (N= 2562)
prepubertal	n= 2907 (71,9%)	n= 1129 (32,4%)	n= 1134 (30,2%)	n= 679 (26,5%)
early pubertal	n= 932 (23.0%)	n= 818 (23,5%)	n= 1783 (47,4%)	n= 697 (27,2%)
mid pubertal	n= 184 (4.5%)	n= 1441(41,3%)	n= 779 (20,7%)	n= 1097 (42,8%)
late pubertal	n= 20 (<1%)	n= 86 (2%)	n= 57 (1,5%)	n= 82 (3%)
postpubertal	n= 2 (<1%)	n= 4 (<1%)	n= 8 (<1%)	n= 7 (<1%)
2 years follow up visit				
	male (N=3970)	female (N= 3378)	male (N= 4095)	female (N= 3299)
prepubertal	n= 1448 (36,5%)	n= 147 (4,4%)	n= 878 (21,4%)	n= 162 (4,9%)
early pubertal	n= 1490 (37,5%)	n= 323 (9,5%)	n= 1715 (41,9%)	n= 360 (10,9%)
mid pubertal	n= 834 (21%)	n= 1601 (47,4%)	n= 1342 (32,8%)	n= 1563 (47,4%)
late pubertal	n= 194 (4,9%)	n= 1249 (37%)	n= 155 (3,8%)	n= 1166 (35,3%)
postpubertal	n= 4 (<1%)	n= 58 (1,7%)	n= 5 (<1%)	n= 48 (1,5%)

Supplementary Table 3: Frequencies of psychopathology measures in the HBN sample.

	male (N=1487)	female (N=784)
Number of diagnoses		
1	n= 384 (25,8%)	n= 202 (25,8%)
2	n= 420 (28,2%)	n= 231 (27,2%)
3	n= 300 (20,2%)	n= 163 (20,8%)
4	n= 175 (11,8%)	n= 98 (12,5%)
5	n= 107 (7,2%)	n= 58 (7,4%)
6	n= 56 (3,8%)	n= 31 (4,0%)
7	n= 31 (2,1%)	n= 16 (2,0%)
8	n= 7 (< 1%)	n= 3 (< 1%)
9	n= 3 (< 1%)	-
10	n= 4 (< 1%)	-
mean (SD)	2.71 (1.62)	2.71 (1.55)
Primary Diagnosis		
ADHD	n= 787 (52,9%)	n= 305 (38,9%)
ASD	n= 144 (9,7%)	n= 25 (3,2%)
Anxiety	n= 190 (12,8%)	n= 196 (25,0%)
Mood	n= 48 (3,2%)	n= 62 (7,9%)
Other Disorder	n= 68 (4,6%)	n= 33 (4,2%)
Other ND	n= 250 (16,8%)	n= 163 (20,8%)
CBCL Total	n= 1269	n= 635
mean (SD)	43,93 (25,99)	42,60 (25,39)

Note: ADHD= Attention Deficit Hyperactivity Disorder, ASD= Autism Spectrum Disorder, ND= Neurodevelopmental Disorder; SD = standard deviation.

Supplementary References

1. Mowinckel, A. M. & Vidal-Piñeiro, D. Visualization of Brain Statistics With R Packages *ggseg* and *ggseg3d*. *Adv. Methods Pract. Psychol. Sci.* 3, 466–483 (2020).
2. Larivière, S. *et al.* The ENIGMA Toolbox: multiscale neural contextualization of multisite neuroimaging datasets. *Nat. Methods* 18, 698–700 (2021).
3. Voldsbekk, I. *et al. Delineating disorder-general and disorder-specific dimensions of psychopathology from functional brain networks in a developmental clinical sample*. http://medrxiv.org/lookup/doi/10.1101/2023.03.31.23288009 (2023) doi:10.1101/2023.03.31.23288009.
