# Supplementary material for: Domain adapted brain network fusion captures variance related to pubertal brain development and mental health
Source: Nat Commun. 2023 Oct 23;14:6698. doi: 10.1038/s41467-023-41839-w (PMC10593774; doi:10.1038/s41467-023-41839-w)
Supplement: Supplementary file 3 — Description of Additional Supplementary Files [file 41467_2023_41839_MOESM3_ESM.pdf]

Description of Additional Supplementary Files:

Supplementary Data 1: Association results for HBN (separate file)

Supplementary Data 2: Association results for ABCD (separate file)
