# Supplementary material for: Domain adapted brain network fusion captures variance related to pubertal brain development and mental health
Source: Nat Commun. 2023 Oct 23;14:6698. doi: 10.1038/s41467-023-41839-w (PMC10593774; doi:10.1038/s41467-023-41839-w)
Supplement: Supplementary file 6 — Reporting Summary [file 41467_2023_41839_MOESM6_ESM.pdf]

# nature portfolio

Corresponding author(s): Dominik Kraft and Tobias Kaufmann

Last updated by author(s): 08/08/2023

# Reporting Summary

Nature Portfolio wishes to improve the reproducibility of the work that we publish. This form provides structure for consistency and transparency in reporting. For further information on Nature Portfolio policies, see our Editorial Policies and the Editorial Policy Checklist.

## Statistics

For all statistical analyses, confirm that the following items are present in the figure legend, table legend, main text, or Methods section.

| n/a | Confirmed | |
|---|---|---|
| ☐ | ☒ | The exact sample size (*n*) for each experimental group/condition, given as a discrete number and unit of measurement |
| ☐ | ☒ | A statement on whether measurements were taken from distinct samples or whether the same sample was measured repeatedly |
| ☐ | ☒ | The statistical test(s) used AND whether they are one- or two-sided<br>*Only common tests should be described solely by name; describe more complex techniques in the Methods section.* |
| ☐ | ☒ | A description of all covariates tested |
| ☐ | ☒ | A description of any assumptions or corrections, such as tests of normality and adjustment for multiple comparisons |
| ☐ | ☒ | A full description of the statistical parameters including central tendency (e.g. means) or other basic estimates (e.g. regression coefficient) AND variation (e.g. standard deviation) or associated estimates of uncertainty (e.g. confidence intervals) |
| ☐ | ☒ | For null hypothesis testing, the test statistic (e.g. *F*, *t*, *r*) with confidence intervals, effect sizes, degrees of freedom and *P* value noted<br>*Give P values as exact values whenever suitable.* |
| ☒ | ☐ | For Bayesian analysis, information on the choice of priors and Markov chain Monte Carlo settings |
| ☒ | ☐ | For hierarchical and complex designs, identification of the appropriate level for tests and full reporting of outcomes |
| ☐ | ☒ | Estimates of effect sizes (e.g. Cohen's *d*, Pearson's *r*), indicating how they were calculated |

*Our web collection on statistics for biologists contains articles on many of the points above.*

## Software and code

Policy information about availability of computer code

Data collection

No data collection was performed by the authors - this is an analysis of publicly available datasets (i.e., ABCD; HBN; PNC)

Data analysis

Magnet resonance imaging (MRI) analyses relied on FreeSurfer (version 7.1.1).

All statistical (association) analyses relied on python 3.7.11 and the following open-source packages:
- pandas (version 1.3.5)
- numpy ( version 1.21.5)
- snfpy (version 0.2.2, https://github.com/rmarkello/snfpy)
- BrainSpace (version 0.1.3)
- scikit-learn (version 1.0.2)
- ADAPT (version 0.4.1)
- neuroCombat (version 0.2.12)
- mapalign (version 0.3.0, https://github.com/satra/mapalign)
- statsmodels (version 0.13.2)
- seaborn (version 0.11.2)
- matplotlib (version 3.5.1
- ENIGMA Toolbox (version 2.0.3)

Additional brain visualizations were performed with:
- ggseg (version 1.6.5) in R (version 4.2.3)

Code is available under: https://github.com/dominikkraft/DomAdapt_BrainNetFusion or https://doi.org/10.5281/zenodo.8223987

For manuscripts utilizing custom algorithms or software that are central to the research but not yet described in published literature, software must be made available to editors and reviewers. We strongly encourage code deposition in a community repository (e.g. GitHub). See the Nature Portfolio guidelines for submitting code & software for further information.

## Data

Policy information about availability of data

All manuscripts must include a data availability statement. This statement should provide the following information, where applicable:

- Accession codes, unique identifiers, or web links for publicly available datasets
- A description of any restrictions on data availability
- For clinical datasets or third party data, please ensure that the statement adheres to our policy

Data used in this study was accessed under data use agreements of the respective study cohorts (ABCD, PNC, HBN). Raw data must not be shared directly by the study authors, but researchers can get access through own data use agreement and use our shared scripts to reproduce the results.

## Human research participants

Policy information about studies involving human research participants and Sex and Gender in Research.

| | |
|---|---|
| Reporting on sex and gender | Statistical analyses across cohorts were stratified for sex and results are reported for males and females seperately.<br>In PNC, sex was obtained from the electronic medical records. In ABCD and HBN sex is defined as biological sex assigned at birth. |
| Population characteristics | This study includes data from three publicly available datasets:<br>For the PNC sample, we included data from N=1594 healthy individuals (females= 834, age: M= 14.95, SD= 3.69).<br>For the HBN sample, we used data from N= 389 healthy individuals (females= 162, age: M= 10.45, SD= 3.81) as held-out data for model building.<br>For the ABCD sample, we used data from N= 3984 healthy individuals (females= 2027, age: M= 9.95, SD= .63) as held-out data for model building.<br><br>Model testing was performed on N=7776 healthy subjects (females= 3587, agebaseline: M= 9.90, SD= .62; agefollow-up: M= 11.90, SD= .65) from the ABCD study as well as on N=2271 (females= 784, age: M= 10.43, SD= 3.45) patients (i.e., subjects with at least one clinical diagnoses) |
| Recruitment | Detailed information can be retrieved from the original manuscripts describing the respective studies:<br>ABCD: see Garavan et al. (2018): doi https://doi.org/10.1016/j.dcn.2018.04.004<br>PNC: see Satterwhaite et al. (2016): doi 10.1016/j.neuroimage.2015.03.056<br>HBN: see Alexander et al. (2017): doi 10.1038/sdata.2017.181 |
| Ethics oversight | PNC: institutional review boards (IRB) of the University of Pennsylvania and the Children`s Hospital of Philadelphia<br>ABCD: approved by either the local site Institutional Review Board (IRB) or by local IRB reliance agreements with the central IRB at the University of California<br>HBN: Chesapeake IRB |

Note that full information on the approval of the study protocol must also be provided in the manuscript.

# Field-specific reporting

Please select the one below that is the best fit for your research. If you are not sure, read the appropriate sections before making your selection.

☒ Life sciences ☐ Behavioural & social sciences ☐ Ecological, evolutionary & environmental sciences

For a reference copy of the document with all sections, see nature.com/documents/nr-reporting-summary-flat.pdf

# Life sciences study design

All studies must disclose on these points even when the disclosure is negative.

| | |
|---|---|
| Sample size | We included as much MRI data as we can gather for the three individual samples (i.e., ABCD; HNB; PNC). Sample size is thus based on data availability |
| Data exclusions | Data exclusion was based on data availability, i.e., participants with missing data, either for the imaging or association analyses were removed for further analyses. |
| Replication | Model building was performed in held-out data from the ABCD and HBN sample, whereas model testing was performed on unseen data from the respective datasets, i.e., we externally validated our model in two timepoints from the ABCD study and in an additional sample of patients |

| | |
|---|---|
| | (HBN) |
| Randomization | Randomization does not apply to this secondary analyses of existing datasets. |
| Blinding | Blinding does not apply to this secondary analyses of existing datasets. |

# Reporting for specific materials, systems and methods

We require information from authors about some types of materials, experimental systems and methods used in many studies. Here, indicate whether each material, system or method listed is relevant to your study. If you are not sure if a list item applies to your research, read the appropriate section before selecting a response.

## Materials & experimental systems

| n/a | Involved in the study |
|---|---|
| ☒ | ☐ Antibodies |
| ☒ | ☐ Eukaryotic cell lines |
| ☒ | ☐ Palaeontology and archaeology |
| ☒ | ☐ Animals and other organisms |
| ☒ | ☐ Clinical data |
| ☒ | ☐ Dual use research of concern |

## Methods

| n/a | Involved in the study |
|---|---|
| ☒ | ☐ ChIP-seq |
| ☒ | ☐ Flow cytometry |
| ☐ | ☒ MRI-based neuroimaging |

# Magnetic resonance imaging

## Experimental design

| | |
|---|---|
| Design type | structural MRI |
| Design specifications | n.a. |
| Behavioral performance measures | n.a. |

## Acquisition

| | |
|---|---|
| Imaging type(s) | T1 weighted |
| Field strength | 3T or 1.5T |
| Sequence & imaging parameters | Detailed information can be retrieved from the original manuscripts describing the respective studies:<br>ABCD: see Casey et al. (2018): doi 10.1016/j.dcn.2018.03.001<br>PNC: see Satterwhaite et al. (2016): doi 10.1016/j.neuroimage.2015.03.056<br>HBN: see Alexander et al. (2017): doi 10.1038/sdata.2017.181 |
| Area of acquisition | whole brain |

Diffusion MRI ☐ Used ☒ Not used

## Preprocessing

| | |
|---|---|
| Preprocessing software | ABCD: preprocessed data was obtained from the ABCD data respository, see 10.1016/j.neuroimage.2019.116091 for details on preprocessing.<br>PNC / HBN: We employed a centralized and harmonized processing protocol including automated surface-based morphometry using Freesurfer 7.1.1 (recon-all) |
| Normalization | We used standard procedure according to 'recon-all' |
| Normalization template | fsaverage |
| Noise and artifact removal | - |
| Volume censoring | - |

## Statistical modeling & inference

| | |
|---|---|
| Model type and settings | We used Elastic Net Regression with an instance based supervised domain adaptation (Transfer AdaBoost for Regression) to learn mappings between raw MRI features (brain area, volumne) and the first embedding derived from diffusion map embedding on a fused network (similarity network fusion). Predicted embedding scores for unseen test data was used for |

association analyses (linear models) with external variables (i.e., puberty scores, sum of diagnoses as mental health proxy measure, Child Behavior Checklist Total Score as measure of dimensional psychopathology)

Effect(s) tested

We used linear models to assess the effect on puberty and mental health on the embedding score or the delta embedding (only for longitudinal data). Linear models were stratified for sex and controlled for 'site' and 'age'. Linear models for puberty in the ABCD sample were repeated with BMI, race/ethnicity & SES as covariates. For longitudinal models, change scores of covariates (e.g., delta age) were introduced if applicable. We provide beta coefficients and exact p-values for all models.

Specify type of analysis: ☐ Whole brain ☒ ROI-based ☐ Both

Anatomical location(s): Desikan-Killiany (68 regions)

Statistic type for inference (See Eklund et al. 2016)

-

Correction

Bonferroni

## Models & analysis

| n/a | Involved in the study |
|---|---|
| ☒ | ☐ Functional and/or effective connectivity |
| ☒ | ☐ Graph analysis |
| ☐ | ☒ Multivariate modeling or predictive analysis |

Multivariate modeling and predictive analysis

Brain volume and area from the Desikan-Killiany atlas were used to construct fused similarity networks with snfpy (version 0.2.2, https://github.com/rmarkello/snfpy). On the final fused matrix we performed diffusion map embedding to derive low-dimensional representations of the imaging data using BrainSpace (version 0.1.3). Diffusion map embedding is a non-linear dimensionality reduction technique that projects the raw data onto dimensions (i.e., embeddings) that encode the primary axes of between-subject similarity. The resulting embeddings are unitless and subjects can be localized according to their inter-subject similarity along these dimensions. For further analyses the first embedding was used, as it captures the highest variance akin to PCA.

For our machine learning framework we then trained an Elastic Net in scikit-learn (version 1.0.2) to learn the mappings between the raw feature space (i.e., area and volume MRI data, each with shape 1594 x 34 after averaging features across both hemispheres) and the first brain embedding. Since our goal was to maximize out of sample generalizability, we 1) trained the model with default parameters (l1_ratio = 0.5 balancing L1 and L2 norm regularization, alpha= 1.0 which tunes the overall penalty strength) aiming at minimizing overfitting to the training set and 2) utilized an instance-based supervised domain adaptation (Transfer AdaBoost for Regression; TrAdaBoostR2) implemented in ADAPT (version 0.4.1). TrAdaBoostR2 combines a source (PNC) and target data set into a single set and performs reverse boosting in which weights of the source instances poorly predicted decrease at each iteration while the ones of the target instances increases, thus shifting the relative importance towards the target set. Thus, the algorithm makes use of those source instances that are similar to the target domain and "ignores" the ones that are more dissimilar. Since increasing the boosting iterations may lead to overfitting, the algorithm per default uses the weighted median of the last N/2 iterations for prediction. To avoid data leakage, we used held-out data from the ABCD and HBN: For the ABCD data we used N= 3984 (females= 2027, age: M= 9.95, SD= .63) children for which only baseline imaging data was available at release 4.0. In the HBN sample we used imaging data from a healthy sample of N=389 (females= 162, age: M= 10.45, SD= 3.81) for which no primary diagnosis was reported. Of note, for the latter we did pool subjects with the label 'no diagnoses' either based on a complete or aborted evaluation. For both datasets we used brain volume and area from the Desikan-Killiany atlas.
